# Biomechanical study of the stiffness of the femoral locking compression plate of an external fixator for lower tibial fractures

**DOI:** 10.1186/s12891-023-06150-1

**Published:** 2023-01-18

**Authors:** Huan Su, Siyang Zhong, Tianyong Ma, Weidong Wu, Yihong Lu, Dewei Wang

**Affiliations:** 1 Second Department of Orthopedics, Fifth Affiliated Hospital of Zunyi Medical University, No. 1439, Zhufeng Avenue, Doumen District, Zhuhai, 519100 China; 2 Zunyi Medical University Zhuhai Campus, No. 368, Jinwan Road, Jinwan District, Zhuhai, 519041 China

**Keywords:** Distal tibial fractures, External fixator, Locking compression plate, Biomechanical analysis

## Abstract

**Background:**

A locking compression plate (LCP) of the distal femur is used as an external fixator for lower tibial fractures. However, in clinical practice, the technique lacks a standardized approach and a strong biomechanical basis for its stability.

**Methods:**

In this paper, internal tibial LCP fixator (Group IT-44), external tibial LCP fixator (Group ET-44), external distal femoral LCP fixator (Group EF-44, group EF-33, group EF-22), and conventional external fixator (Group CEF-22) frames were used to fix unstable fracture models of the lower tibial segment, and anatomical studies were performed to standardize the operation as well as to assess the biomechanical stability and adjustability of the distal femoral LCP external fixator by biomechanical experiments.

**Results:**

It was found that the torsional and flexural stiffnesses of group EF-44 and group EF-33 were higher than those of group IT-44 and group ET-44 (*p* < 0.05); the flexural stiffness of group EF-22 was similar to that of group IT-44 (*p* > 0.05); and the compressive stiffness of all three EF groups was higher than that of group ET-44 (*p* < 0.05). In addition, the flexural and compressive stiffnesses of the three EF groups decreased with the decrease in the number of screws (*p* < 0.05), while the torsional stiffness of the three groups did not differ significantly between the two adjacent groups (*p* > 0.05). Group CEF-22 showed the highest stiffnesses, while group ET-44 had the lowest stiffnesses (*P* < 0.05).

**Conclusions:**

The study shows that the distal femoral LCP has good biomechanical stability and adjustability and is superior to the tibial LCP as an external fixator for distal tibial fractures, as long as the technique is used in a standardized manner according to the anatomical studies in this article.

## Background

The anatomical location of the tibia is superficial, and the soft tissue coverage is weak. Open fracture easily occurs under high-energy injury factors and is often accompanied by severe soft tissue injury. In addition, the fracture prognosis is poor, and complications such as bone nonunion, infection and limb necrosis can even occur [1–5]. In this case, the principal clinical approach is to treat the fracture with an external fixator in phase I and then an internal fixator in phase II to promote fracture healing [6–8]. The external fixator frame is less disruptive to the soft tissues and blood flow near the fracture site, making it more convenient for the fracture to heal after surgery, and it also has the advantages of being simple, quick and easy to adjust [9–11]. Therefore, external fixators are typically used as a temporary or definitive treatment for open fractures or closed fractures with poor soft tissue conditions. However, external fixators have disadvantages such as heavy frames, pin track infection, and loose frame structures. In addition, the method of transarticular fixation with external fixators also has the disadvantage of easily leading to irreversible joint stiffness and dysfunction [12–14]. While internal fixation techniques are characterized by reliable fixation and accurate reduction, internal fixation surgery for such fractures increases the risk of infection and severely disrupts the blood supply to the periosteum, resulting in serious complications such as skin necrosis, osteomyelitis, delayed fracture healing or nonunion, and potential amputation [15–17]. Therefore, there is no satisfactory fixation method for the treatment of open fractures of the lower tibia or closed tibial fractures with severe soft tissue injuries.

Marti et al. were the first to use an internally fixed tibial anatomic locking splint as an external fixator in 1984 to treat open fractures, infected bone discontinuity, septic arthritis and other related conditions [15, 18]. Subsequent finite element analysis and biomechanical studies on the stability of LCP external fixators for tibial fractures have shown that LCP has good biomechanical stability as an external fixator [19–26]. At the same time, some scholars have also applied LCP in clinical practice to treat open tibial fractures and have obtained more satisfactory results in clinical practice [27–33]. However, the distal femoral LCP is designed for internal fixators, and there are certain ethical issues in using it as an external fixator in the clinic, which limits its wide application [24, 34].

The use of femoral LCP external fixation for the treatment of open fractures of the lower tibia or closed tibial fractures with severe soft tissue injury has the advantages of reliable fixation, small size, small injury, short operation time, good efficacy, low cost, and no need for a second operation [24]. This fixation modality can be used for both temporary and ultimate fixation and has other advantages. However, there is a lack of standardized clinical practice and systematic biomechanical studies on the proper use of this technique. Therefore, this study was conducted to standardize the technical aspects of the surgical operation through anatomical studies and to create conditions for clinical treatment. In this study, a synthetic tibial bone model was prepared to study the biomechanical stability and adjustability of an LCP external fixator of the distal femur through biomechanical experiments, which provides a theoretical basis for clinicians to treat open fractures of the lower tibia or closed tibial fractures with severe soft tissue injuries.

## Methods

### Anatomical studies of the distal femur LCP and tibia

**Fig. 1:**
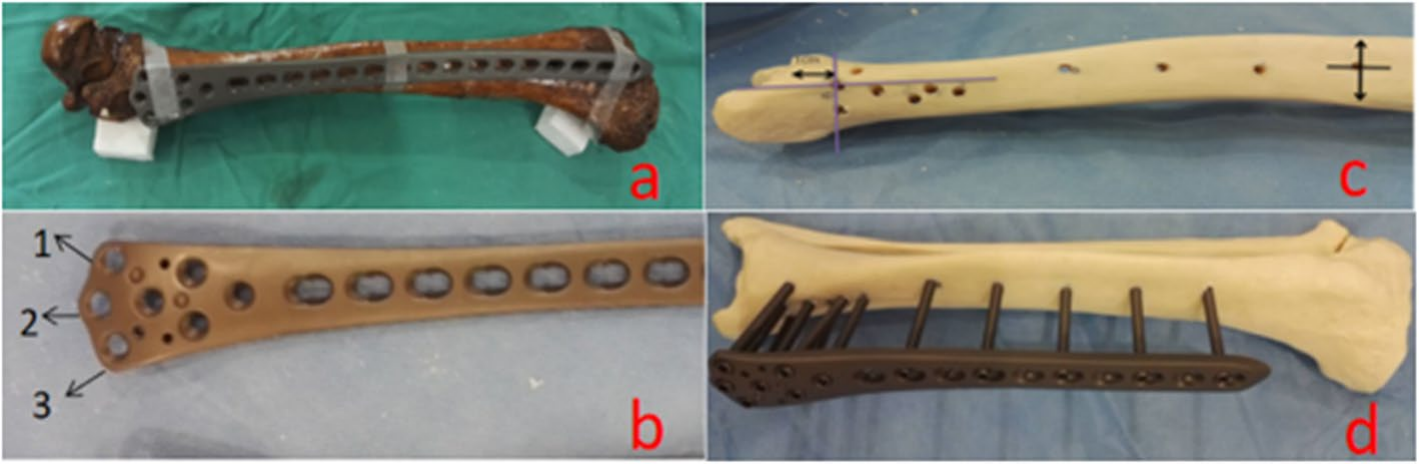
Anatomical study of the distal femoral LCP and the tibia: **a** The distal femoral LCP is morphologically similar to the tibia and can be affixed to the anteromedial side of the tibia; **b** 2 is the most distal hole of the distal femoral LCP; **c** The placement of the LCP was determined by identifying the two most distal and most proximal holes of the LCP; **d** Specimen of the LCP with the tibia for fixation

It has been reported that placing the LCP on the anteromedial or medial side of the tibia results in higher biomechanical stability [20, 24]. Therefore, in this paper, the distal femur LCP was placed on the anteromedial side of the tibia. By using a synthetic tibial bone model as the material, the anatomical morphology of the tibia, the morphology of the distal femoral LCP and the distribution of the locking nail were studied to determine the best fixation method for the external fixator of the lower tibial fracture with the distal femoral LCP (Fig. 1): 1. The screw was locked through the most distal hole of the LCP to determine the distal positioning of the LCP, which included determining its distance from the articular surface, the anterior–posterior position of the tibia and the angle of the nail, to identify the distal position. The best three-dimensional coordinates of the locating nail were determined by locking the screw through the most distal hole of the LCP to identify the proximal positioning, thus determining the best coordinates in the tibial stem. 3. The distal and proximal locating nails were used to determine the LCP position so that the locking screws of all other holes of the LCP could be fixed to the tibia, and the relationship between the position of these locking nails and the entrance and exit of the bone surface and the neurovascular was observed. The above experiments were used to determine the optimal positioning points and coordinates of the distal and proximal locating nails of the LCP, which laid the foundation for the standardized operation of this procedure.

### Preparation of the distal tibial fracture model

Ninety artificial composite tibial models were randomly divided into six groups (15 models in each group, randomly divided into three equal parts, 5 models for each group were subjected to axial compression, three-point bending and axial torsion tests): a tibial LCP internal fixator group (far 4 near 4, group IT-44), a tibial LCP external fixator group (far 4 near 4, group ET-44), a conventional external fixator frame group (far 2 near 2, group CEF-22), and three distal femur LCP external fixator groups (far 4 near 4—group EF-44, far 3 near 3—group EF-33 and far 2 near 2—group EF-22, respectively) (two values of number of screws at the distal and proximal fracture ends).

**Fig. 2:**
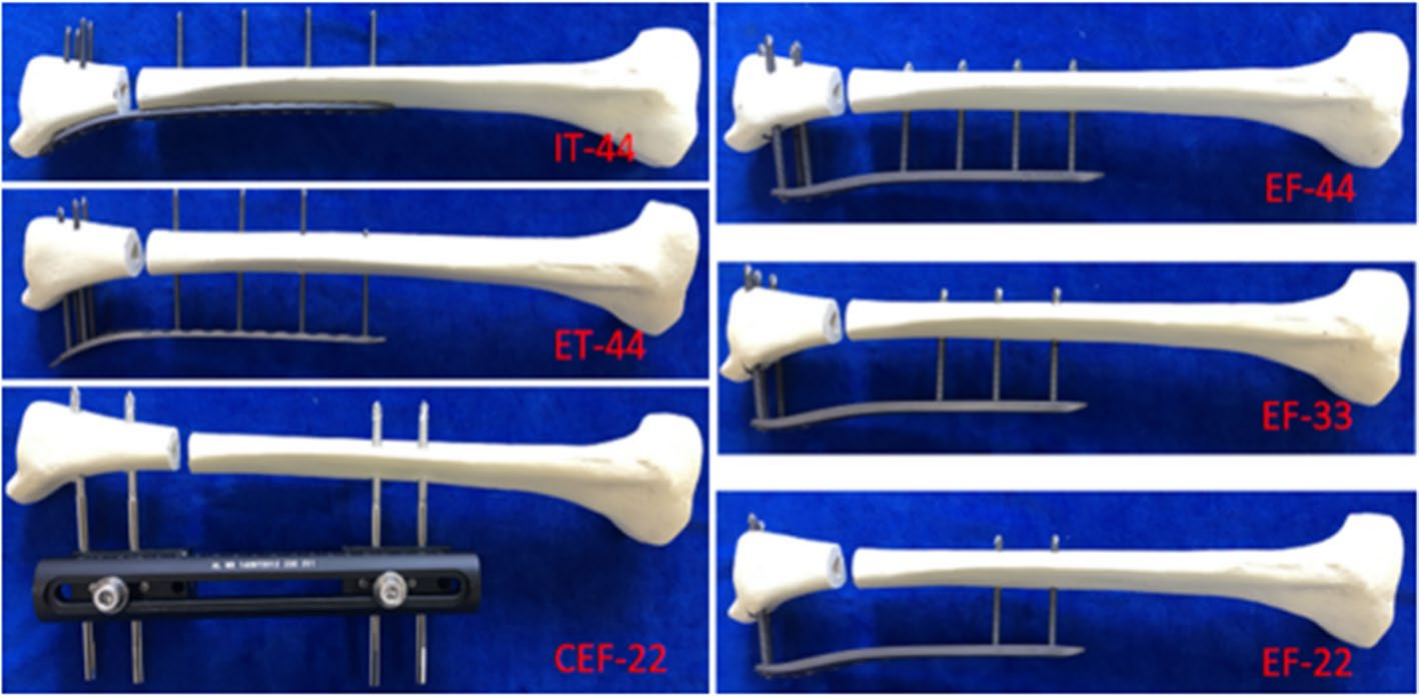
Examples of different specimen models. IT-44: tibial LCP internal fixator group (far 4 near 4); ET-44: tibial LCP external fixator group (far 4 near 4); CEF-22: conventional external fixator frame group (far 2 near 2); EF-44, EF-33, EF-22: three femur LCP external fixator group (far 4 near 4, far 3 near 3 and far 2 near 2, respectively) two values of the number of screws at the distal and proximal fracture ends)

Detailed procedures for each group were as follows:

The IT-44 group was fixed with an ipsilateral 3.5-mm 12 + 8-hole distal tibial LCP internal fixator (Trauson Medical Instrument Co., Ltd., Jiangsu, China) by means of standard pure titanium 3.5 mm locking screws with proximal and distal screw distributions of 1, 4, 7, 10 and 7, 18, 19, 20, respectively (Fig. 2 IT-44). The ET-44 group was fixed with an ipsilateral 3.5-mm 12 + 8-hole distal tibial LCP external fixator at 30 mm from the bone surface (Trauson Medical Instrument Co., Ltd., Jiangsu, China) with proximal and distal screw distributions of 1, 4, 7, 10 and 7, 18, 19, 20, respectively (Fig. 2 ET-44). All three EF groups were fixed with a contralateral 5-mm 8 + 7-hole distal tibial LCP at 30 mm from the bone surface (Trauson Medical Instruments Co., Ltd., Jiangsu, China) with standard pure titanium 5.0 mm locking screws for fixation. However, the distribution of the screws was not consistent among the three groups: 1, 3, 5, 7 and 12, 13, 14, 15 for proximal and distal screws in group EF-44; 1, 3, 5 and 13, 14, 15 for proximal and distal screws in group EF-33; 1, 3 and 14, 15 for proximal and distal screws in group EF-22 (Fig. 2 EF-44, EF-33, EF-22). The CEF-2 group was externally fixed using the protocol of an inlay external fixator bracket-slide bar (Trauson Medical Devices Co., Ltd., China), which was fixed by metal bone pins with straight lateral clamping nails at a distance of 30 mm from the bone surface (Fig. 2 CEF-22). All the screws were tightened with a torque screwdriver. The working length of each structure is as follows: the distance between the screws on both sides of the fracture ends was 6 cm in the IT-44, ET-44, and EF-44 groups, while the distance between the screws on both sides of the fracture ends was 10 cm, 14 cm, and 14 cm in the EF-33, EF-22, and CEF-22 groups, respectively. According to the international standard [20, 23, 24], after the tibial model was fixed according to the corresponding fixation method, the unstable lower tibial fracture model was prepared by transection at 50 mm on the ankle joint surface, causing a 10 mm defect area (Fig. 2).

### Biomechanical experiments

**Fig. 3:**
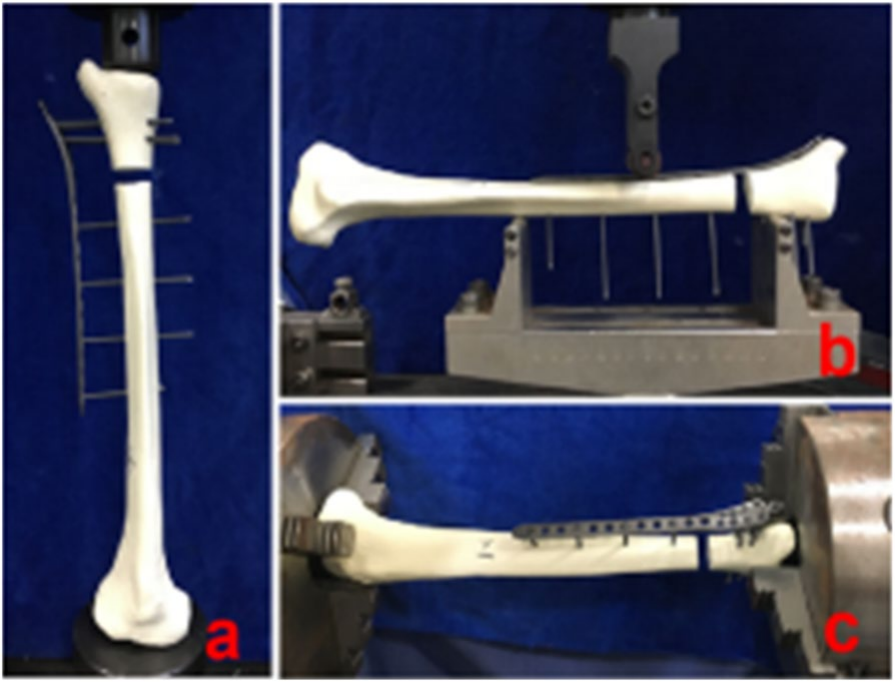
Assembly diagram of each group: **a** Axial compression test; **b** Three-point bending test; **c** Torsion test

The electronic universal testing machine (No.: CMT5105/TC-A-05, provided by Transon Medical Instrument Co., Ltd., Jiangsu, China) and electronic torsion testing machine (No.: TTM202A/TC-A-65, provided by Transon Medical Instrument Co., Ltd., Jiangsu, China) were used for biomechanical testing (testing was performed at the Yangtze River Testing Center of Transon Medical Instrument Co., Ltd. The tests included the three-point bending test, axial compression test and torsion test. Tests were conducted to compare the stiffness of different fixation methods against bending, compression and rotation and to screen out the biomechanical stability and the best fixation method for clinical application. Each group of specimens was assembled and tested in the following manner (Fig. 3):

- Axial compression test: The specimen was placed vertically, and the load was applied at a speed of 5 mm/min until the upper and lower surfaces of the bone defect were in contact. The test was then stopped, and the load‒displacement curve was recorded. Five specimen tests were conducted in each group.
- Three-point bending test: The specimen was placed horizontally, with support span L = 170 mm. The load was applied at a rate of 5 mm/min until the specimen yielded. Testing was then stopped, and the load‒displacement curve was recorded for each group of five specimens tested.
- Torsion test: A three-jaw chuck was used to hold both ends of the specimen, lock it, and apply torque at 1 r/min until the specimen yielded significantly. The test was stopped, and the torque-torsion angle curve was recorded. Five specimen tests were conducted per group.

### Statistical analysis

The load‒displacement curves for each group were obtained by integrating the load‒displacement curves for each group of 5 samples. The stiffness was determined as the slope of the linear part of the curve using Excel [24]. The experimental data are expressed as the mean ± standard deviation (mean ± SD) and were statistically analyzed using the SPSS 26.0 software package. One-sided factorial ANOVA and post hoc multiple tests (LSD, Tamhane T2) were performed on the data; *p* < 0.05 was considered a statistically significant difference.

## Results

### Anatomical studies

In this study, the morphology of the distal femoral LCP was found to be very similar to that of the contralateral tibia (Fig. 1a) and could be affixed to the anteromedial surface of the tibia. In this experiment, the distal femoral LCP was externally placed on the anteromedial side of the contralateral tibial fracture model by first locating the position of distal foramen 2 (Fig. 1b)—the intersection of a 10 mm plumb line on the articular surface and the horizontal line of the anterior border of the medial ankle (Fig. 1c)—and then locating the position of the proximal foramen—the midpoint of the anterior and posterior tibial crest (Fig. 1c). The LCP position was fixed by locating the nail through the distal and proximal holes, and then the corresponding locking screws were driven in the other holes (Fig. 1d). Based on the results of the anatomical study here, placing the LCP on the anteromedial or medial side of the tibia can achieve good stability.

### Biomechanical testing

**Fig. 4:**
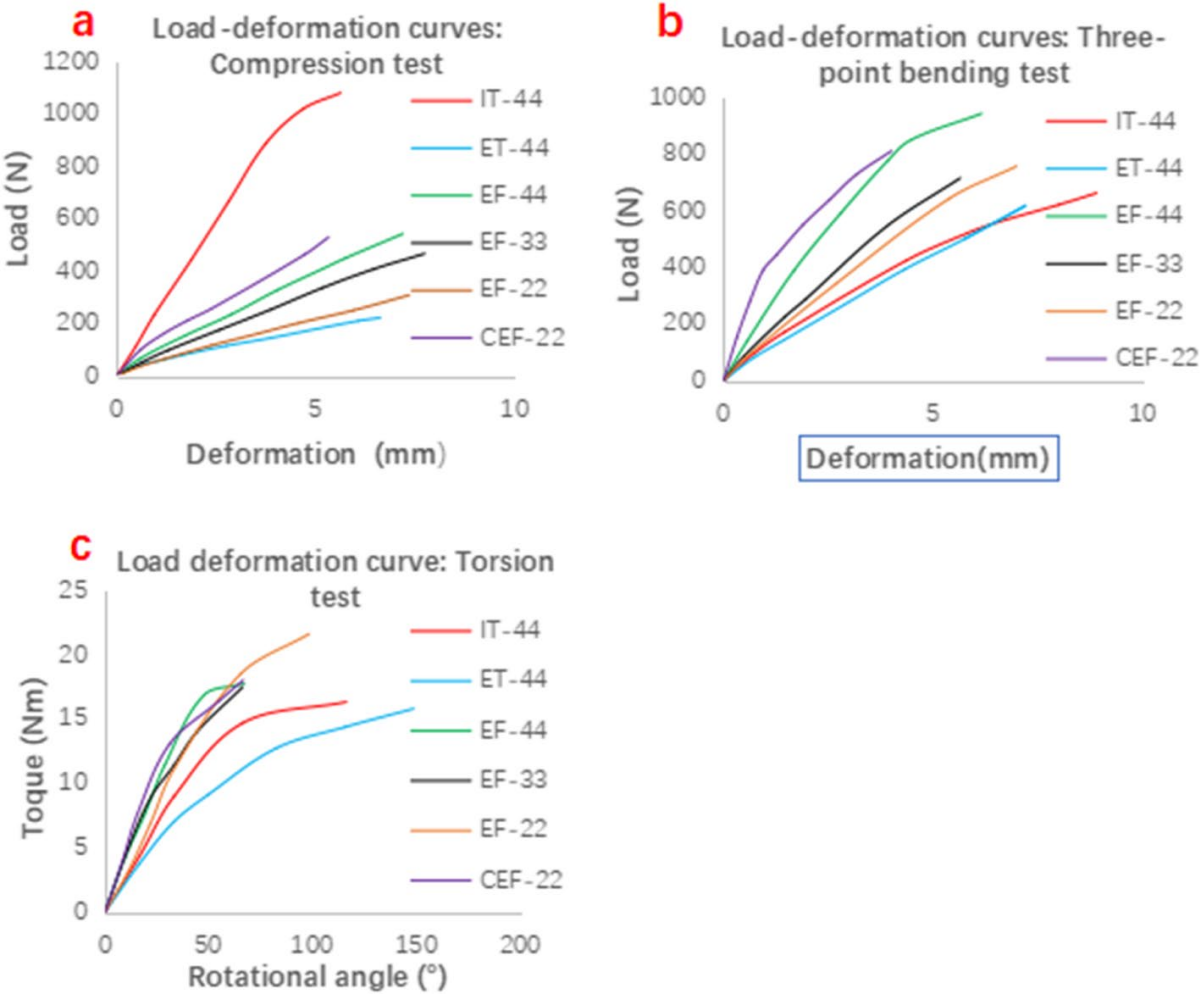
Load deformation curves in the different models. **a** Compression test; **b** Three-point bending test; **c** Toison test. The deformation curves were plotted as a red line for the IT-44 model, a blue line for the ET-44 model, a green line for the EF-44 model, a black line for the EF-33 model, an orange line for EF-22 model and a purple line for the CEF-22 model

The load‒displacement curves of the axial compression tests for each group of specimens (Fig. 4a) showed that the yield point was reached at a load of approximately 600 N for all five groups except the samples of group IT-44. However, the three EF groups and the CEF-22 group did not yield at the yield load of group ET-44. Under the same load, group IT-44 samples deformed the least, indicating that group IT-44 samples had the highest shaft compressive stiffness. In addition, the compressive stiffness of the samples in the three EF groups was higher than that in group ET-44. The load‒displacement curves of the specimens in each group were obtained in the three-point bending test (Fig. 4b). The specimens in the EF-44 and CEF-22 groups did not bend when loaded to 800 N, while the specimens in the other four groups did. The specimens in the three EF groups remained unbent when the IT-44 and ET-44 groups were bent. The torsion-torque curves of each group of specimens (Fig. 4c) showed that the torsion angles of the specimens in group CEF-22, group EF-44 and group EF-33 were similar in the range of applied torque of 0–5 Nm, while the torsion angles of group EF-22, group ET-44 and group IT-44 were similar; however, under the same torque, the torsion angles of group CEF-22, group EF-44 and group EF-33 were smaller than those of group ET-44 and group IT-44.

One-way ANOVA and post hoc multiple testing of the data showed that there were significant differences in flexural, compressive and rotational stiffness between the six groups (two-by-two comparison, at least two groups, *p* = 0.00 < 0.01), which was statistically significant (see Table 1).

- (1) In terms of axial compression stability, the compressive stability of each group from high to low was IT-44 > CEF-22 > EF-44 > EF-33 > EF-22 > ET-44, and there was a significant difference between the six groups compared with each other (*p* < 0.01).
- (2) In terms of flexural stability, all three EF groups were higher than the IT-44 and ET-44 groups (*p* < 0.01), the EF-44 group was higher than the EF-33 group (*p* = 0.005 < 0.01), and the CEF-22 group was higher than all other groups (*p* = 0.00 < 0.001). There was a significant difference between the two comparisons; the IT-44 group was higher than the ET-44 group (*p* > 0.05), and the EF-33 group was higher than the EF-22 group (*p* > 0.05).
- (3) In terms of anti-torsional stability, group EF-44 and group EF-33 were higher than group IT-44 and group ET-44 (*p* < 0.01), group ET-44 was lower than all other groups (*p* < 0.05), group CEF22 was higher than group IT-44, group ET-44 and group EF-22 (*p* < 0.05), the difference was significant; group EF-44 and group EF-33 were not significantly different from group CEF22 (*p* > 0.05); three EF groups were compared between two adjacent groups (*p* > 0.05) and the difference was not statistically significant; group EF-44 was compared with group EF-33 and the difference was statistically significant.

**Table 1:** Comparison of compression stiffness, flexural stiffness and torsional rigidity in the different models (mean ± SD)

Group	Compression test (N/mm)	Three-point bending test (N/mm)	Torsion test (Nm/°)
IT-44	252.53 ± 2.61	92.78 ± 1.24	0.34 ± 0.01
ET-44	34.03 ± 1.22	85.72 ± 3.24	0.24 ± 0.01
CEF-22	125.51 ± 6.94	375.79 ± 4.91	0.51 ± 0.10
EF-44	78.42 ± 1.51	202.36 ± 17.02	0.48 ± 0.02
EF-33	56.91 ± 1.09	135.18 ± 11.21	0.46 ± 0.02
EF-22	40.00 ± 0.40	122.72 ± 7.84	0.39 ± 0.04
F	3409.96	625.23	23.60
P	= 0.00 < 0.01	= 0.00 < 0.01	= 0.00 < 0.01

## Discussion

The stability of the LCP plays a major role in determining the healing process of complex fractures. Studies have shown that the main factors affecting LCP stability include plate length and thickness, number and distribution of screws, plate length, distance between the plate and the bone surface, and LCP placement [23, 24]. Kanchanomai et al. [23] suggested that the structural stability of the LCP for external fixators of the tibia in stable fractures (fracture gap of 1 mm) allows patients to undergo early partial weight-bearing after surgery, whereas in unstable fractures (fracture gap of 5–10 mm), the fixation stability is lower than the former, and the failure rate of early weight-bearing external fixators is higher.

In an experiment investigating the relationship between the distance between the LCP and fracture healing, it was shown that the distance between the LCP and the bone surface did not change much in the structural stability of the LCP for distances less than 2 mm; when the distance was greater than 5 mm, the axial compression strength of the LCP decreased by 63%, and its structural stability was greatly reduced [22]. Ching-Hou Ma et al. treated 52 open tibial fractures using the tibial LCP as an external fixator for 38 months of follow-up [25]. They performed both static axial compression and torsion tests to assess the strength of this fixation technique. The results indicated that the distal tibial LCP external fixator was not as strong as standard internal locking plate constructs, thus necessitating further biomechanical studies to improve structural strength. It has been found that LCP external fixator stability decreases with increasing distance and provides good biomechanical stability only when the limiting distance between the LCP and bone is less than 30 mm [20, 24]. Therefore, the farthest distance between the bone and LCP was selected as 30 mm in this study. The distance between the bone surface and the plate significantly affects the stiffness of the LCP, and in this paper, by studying the IT-44, ET-44 and EF-44 groups, the results showed that the stability of the LCP decreases with increasing distance, which is consistent with the results of related reports [24].

Liu et al. found that increasing the screw diameter and plate size significantly improved torsional stiffness but had little effect on compression stiffness. This confirms that the biomechanical stability of the distal femoral LCP is superior to that of the distal tibial LCP and is more suitable as an external fixator for the treatment of lower tibial fractures, which is consistent with the results of this study [24]. In this study, by changing the size of the plate and the diameter of the screw, it was found that the torsional and bending stability of group EF-44 was higher than that of group IT-44 and group ET-44, and group IT-44 was higher than that of group ET-44. In terms of compression resistance, group IT-44 had a higher compression stiffness than group EF-44 because it was attached to the bone surface, while group ET-44 was the worst. Although the axial compression stiffness of the distal femoral LCP external fixator was insufficient, the axial compression stiffness was higher than that of the distal tibial LCP external fixator, and the bending and torsional stiffness were higher than those of the distal tibial LCP external fixator and distal tibial LCP internal fixator. Bottlang [35] used a locking plate to fix femoral fractures 1 mm away from the bone surface to investigate the effect of its structural stiffness on fracture healing, and it was found that this fixation method made the axial micromotion of the fracture and could stimulate osteogenesis at the fracture site, thereby promoting fracture healing. According to the biomechanical properties of the structure in this study, the LCP of the distal femur can perform elastic fixation at the fracture site, and we speculate that this fixation method can produce axial micromotion at the fracture site and stimulate osteogenesis at the fracture site to promote fracture healing [20, 24, 34, 36–38].

Studies have found that the LCP can also achieve similar torsional and flexural stiffness with the selection of a smaller number of screws for fixation [39–42]. All three EF groups had higher flexural stiffness than group IT-44 and group ET-44, group EF-44 and group EF-33 had higher flexural stiffness than group IT-44 and group ET-44, and group EF-22 had a similar torsional resistance to group IT-44. The number of plate screw fixations had a significant effect on the stability of the plate [41, 42]. A study comparing the three EF groups showed that in terms of compression resistance and flexural stability, the stability decreased with the decrease in the number of screws, proving that the distal femoral LCP external fixator has good biomechanical stability and adjustability. However, there was no statistically significant difference in torsional stiffness between the two adjacent groups of the three EF groups, proving that a small increase in the number of screws would not significantly increase the torsional stiffness. The elastic fixation of the steel plate can produce axial micromovement at the fracture end, and the stress stimulation of axial micromovement can promote fracture healing [20, 24, 34–37]. LCP external fixation of the distal femur has biomechanical adjustability. During fracture healing, the LCP fixation strength is gradually reduced by gradually decreasing the number of screws, thereby reducing stress shading and generating axial micromotion at the fracture to stimulate osteogenesis, thereby promoting fracture healing and shortening the time to healing.

For the first time, in a biomechanical study of the stability of fixators for lower tibial fractures, this paper presents a comprehensive study of conventional external fixators, internal tibial LCP fixators, external tibial LCP fixators, and external distal femoral LCP fixators. Hoenig, Yang, and Liu et al. found that distal femoral LCP external fixators had approximately the same stiffness as standard plates or Ilizarov fixators, consistent with the experimental findings in this paper [24, 43, 44]. The biomechanical stability of the steel plate clearly influences the healing of the fracture, and fixator structures that are either too stiff or too flexible will hinder fracture healing and lead to delayed healing or bone nonunion [24, 45]. In this study, the CEF-22 group was found to have the best biomechanical stability, but most of the external fixators were unstable, and a few were fixed with too much strength and would produce stress masking. Both of these conditions can lead to bone nonunion and delayed healing, which are also common in clinical practice. Moreover, prolonged fixation of the external fixator across the joint can lead to functional stiffness of the joint. In addition, the large size of the external fixation frame can cause inconvenience and psychological problems for patients. The biomechanical results of this study showed that tibial LCP external fixation had the worst compressive, flexural and rotational stability, which is consistent with the reported results [24]. The stability against compression, bending and rotation of the distal femoral LCP external fixator was higher than that of the distal tibial LCP external fixator, but the axial compressive stability of the distal femoral LCP external fixator was lower than that of the distal tibial LCP internal fixator. This is because the closer the LCP distance, the more structural the splice plate is [24]. However, its moderate flexibility may promote fracture healing. Therefore, for the selection of an external fixator for the treatment of lower tibial fractures, the distal femoral LCP is superior to the distal tibial LCP.

**Fig. 5:**
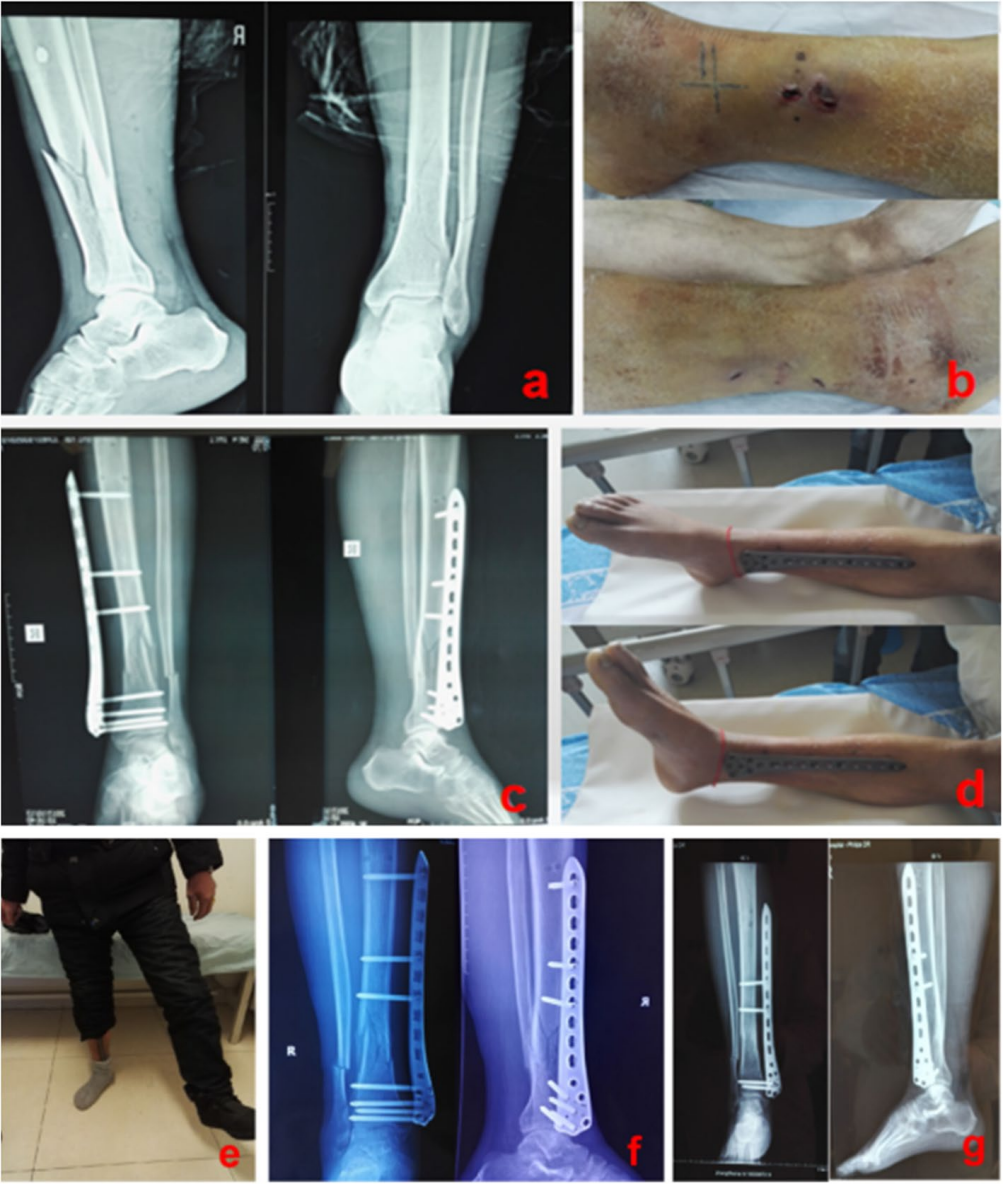
Patient with a comminuted fracture of the lower tibia and severe soft tissue contusion (**a**, **b**); closed reduction with external fixator of the distal femur with LCP (**c**); functional ankle exercises can be performed on the second postoperative day (**d**). Full weight-bearing at 2 weeks postperatively (**e**); small amount of bone scab formation at the fracture end at 1.5 months postperatively (**f**); bony healing at 4.5 months postperatively (**g**)

Zhou et al. used a locking compression plate as an external fixator to treat closed distal tibial fractures with soft tissue injury [31]. This trial provided successful cases with high healing rates, comfortable clinical procedures, and good ankle motion; however, this medical treatment has limited indications. Therefore, to increase the clinical relevance of this study, a case of distal femoral LCP as an external fixator for lower tibial fractures is provided. LCP was originally designed for use as an internal fixator, as its use as an external fixator would be unethical and has limited indications [24, 31, 34]. Therefore, according to the purpose of this study, one patient selected for both conventional internal fixator and external fixator treatment was unsuitable. The hospital ethics committee and internal review board approved the study, and informed consent was obtained from this patient. Patient-related information was as follows: Male, 56 years old, crushed by a 2-ton weight of channel steel, closed lower tibial fracture with soft tissue injury (Fig. 5a, b).

According to the results of the above anatomical study, we treated this patient with external fixation with a 5 mm 10-hole LCP (Chuang Sheng Medical Equipment Co., Ltd., Jiangsu, China) of the distal femur, fixed with four and three 5 mm locking screws at the distal and proximal holes of the fracture, respectively. After the patient underwent closed reduction of the fracture with an external LCP fixator of the distal femur (Fig. 5c), functional ankle exercises were performed on the first day (Fig. 5d). Full weight-bearing was possible 2 weeks after surgery (Fig. 5e). Radiographs at 1.5 months postoperatively showed a small amount of bone scab formation at the fracture end (Fig. 5f). The reduction of 3 screws (1 proximal and 2 distal) at 2.5 months postoperatively was found to facilitate fracture healing after reducing the stress shading. Bony healing at 4.5 months postoperatively (Fig. 5g) allowed the removal of the external fixator plate. It can be hypothesized from this case that the external fixator of the femoral LCP has good biomechanical stability and adjustability for fracture healing when the technique is used in a standardized manner according to the anatomical study in this article.

This study concluded that distal femoral LCP promotes fracture healing with several advantages: (1) The soft tissue on the bone surface is the main source of blood supply to the fracture end, and a distal femoral LCP external fixator can reduce stripping and damage to the periosteum. (2) Because of the relatively thick distal femoral LCP plate, there are more screws at the distal end, and its locking screws are thick in diameter and long enough to fix both cortices, which has good stability. (3) As the fracture heals, the reduction in the number of screws not only provides good stability but also reduces stress shielding and allows early functional exercise to promote fracture healing. (4) The contour of the distal femoral LCP is similar to the curvature of the anteromedial tibial side, and the plate can be placed closer to the skin on the anteromedial side. The patient can wear socks and pants normally to cover the plate after surgery, which meets the patient’s aesthetic needs. (5) The distal femoral LCP is made of titanium alloy, which is lightweight, inexpensive, and histocompatible and has high patient acceptance. Moreover, it is easy to remove after surgery to avoid secondary surgical injury.

For lower tibial fractures, the distal femoral LCP is more suitable for use as an external fixator than the tibial LCP and conventional external fixators [24], and this trial confirms this view. Therefore, the distal femoral LCP deserves to be promoted as an external fixator. In this study, the distal femoral LCP was found to have higher axial compression stiffness, torsional stiffness, and flexural stiffness, making it a better choice for external fixator treatment of lower tibial fractures. Although this study showed that the distal femoral LCP external fixator for distal tibial fractures can provide adequate stability, it is important to recognize the limitations of this study: first, we used a composite tibial model and did not use real human bone, which cannot simulate real human weight-bearing. Second, the effect of muscle forces, etc., was not considered, and this experiment was a static test, which cannot simulate the stability of the plate during motion,. Third, there was no further study of the effect of the number and distribution of screws on the stability of the plate. Fourth, there was no assessment of the LCP stability when axial micromovement was produced at the fracture end. Our team will continue to enhance subsequent trials to provide a sufficient basis for the clinical use of distal femoral LCP external fixators for distal tibial fractures.

Despite the shortcomings of this trial, a distal femoral LCP is still recommended for use as an external fixator for the treatment of distal tibial fractures. Considering all the advantages and risks, the indications should be understood and used with caution [24, 31, 32, 34, 38]. However, it is still possible that a distal femoral LCP external fixator can be used as an external fixator for lower tibial fractures. Although this fixation method cannot replace conventional incisional internal fixators, the use of distal femoral LCP external fixators may yield unexpected clinical results in cases of severe soft tissue problems associated with lower tibial fractures, which can cause serious complications with the use of conventional internal fixators and common external fixator frames. This technique embodies both the minimally invasive and fast track surgery (FTS) concepts. It can be used as both temporary fixation and ultimate fixation and is a treatment modality worth promoting.

## Conclusion

The distal femoral LCP is similar in curvature to the tibia, and the LCP is placed parallel to the anteromedial tibia, allowing effective fixation of the lower tibial fracture as long as it is done in a standardized manner according to this study. Distal femoral LCP external fixation has high bending and rotational stiffness but insufficient compressive stiffness. Because this fixation method has the biomechanical characteristics of elastic fixation and adjustability, it can reduce stress shielding at the fracture site and stimulate fracture osteogenesis to promote fracture healing. This study will provide a theoretical basis for the treatment of open fractures of the lower tibia or closed fractures of the lower tibia with severe soft tissue injury. When it is difficult to treat open fractures of the lower tibia or closed fractures with severe soft tissue injuries in clinical practice, the choice of this fixation technique may achieve unexpected therapeutic results.

## Data Availability

The datasets used and/or analyzed during the current study are available from the corresponding author on reasonable request.

## Abbreviations

LCP: Locking compress plates
IT-44: Internal tibia locking plate fixation 44
ET-44: External tibia locking plate fixation 44
EF-33: External femoral locking plate fixation 33
EF-22: External femoral locking plate fixation 22
ANOVA: Analysis of variance
FTS: Fast track surgery
